# Evaluating the Readability of Online Blood Cancer Education Materials Across Different Readability Measures

**DOI:** 10.7759/cureus.58488

**Published:** 2024-04-17

**Authors:** Ashley Shin, Surbhi Banubakode, Sara Taveras Alam, Anneliese O Gonzalez

**Affiliations:** 1 Division of Hematology/Oncology, McGovern Medical School at UTHealth Houston, Houston, USA

**Keywords:** lymphoma, leukemia, blood cancer, readability, physician education materials, patient education materials

## Abstract

Introduction

The National Institutes of Health and the American Medical Association recommend patient education materials (EMs) be at or below the sixth-grade reading level. The American Cancer Society, Leukemia & Lymphoma Society, and National Comprehensive Cancer Network have accurate blood cancer EMs.

Methods

One hundred one (101) blood cancer EMs from the above organizations were assessed using the following: Flesch Reading Ease Formula (FREF), Flesch-Kincaid Grade Level (FKGL), Gunning Fog Index (GFI), Simple Measure of Gobbledygook Index (SMOG), and the Coleman-Liau Index (CLI).

Results

Only 3.96% of patient EMs scored at or below the seventh-grade reading level in all modalities. Healthcare professional education materials (HPEMs) averaged around the college to graduate level. For leukemia and lymphoma patient EMs, there were significant differences for FKGL vs. SMOG, FKGL vs. GFI, FKGL vs. CLI, SMOG vs. CLI, and GFI vs. CLI. For HPEMs, there were significant differences for FKGL vs. GFI and GFI vs. CLI.

Conclusion

The majority of patient EMs were above the seventh-grade reading level. A lack of easily readable patient EMs could lead to a poor understanding of disease and, thus, adverse health outcomes. Overall, patient EMs should not replace physician counseling. Physicians must close the gaps in patients’ understanding throughout their cancer treatment.

## Introduction

Approximately 10% of all cancer diagnoses in the United States are blood cancers, including leukemia, lymphoma, and myeloma [1]. Furthermore, blood cancer accounts for about 42-52% of pediatric cancer diagnoses, the most common of which is leukemia [2]. Cancer patients have a unique set of needs: a cancer diagnosis often disrupts, or even halts, patients’ daily lives. This is especially true for adolescent and young adult cancer patients, in whom physical, emotional, and psychological development and change are abundant. During this time, education about their diagnosis can serve as a coping mechanism for newly diagnosed cancer patients.

As more people turn to the internet for healthcare-related information, the resources available should be at a reading level that most people can easily understand [3]. Younger cancer patients prefer to learn about their diagnosis through the Internet and social media [4]. Based on the National Institutes of Health (NIH) and American Medical Association (AMA) recommendations, public patient education materials (EMs) should be at or under the sixth-grade reading level [5]. Reading levels correspond to the school grade, ranging from elementary school to the graduate school level, within the United States education system. The American Cancer Society (ACS), Leukemia & Lymphoma Society (LLS), and National Comprehensive Cancer Network (NCCN) are nationally recognized organizations that have accurate information on these complex diseases that physicians believe are good resources for patients. In addition, the LLS provides health professional education materials (HPEMs) to serve as resources for healthcare professionals. We hypothesized that physician education materials would be at a higher reading level than patient education materials.

The effectiveness of patient EMs relies on the ease with which the content can be comprehended. Health literacy, an individual's capacity to access and comprehend health information for informed decision-making, holds considerable significance [6]. Individuals with low health literacy, and poor literacy in general, can encounter more difficulties when faced with complex EMs. Risk factors for low health literacy include older age, lower level of education, lower socioeconomic status, and language barriers [7]. Poor readability further negatively affects patients with low health literacy, exacerbating the existing disparities in healthcare. Complex EMs may lead to an incomplete or inaccurate understanding of one's health condition, treatment options, and recommended actions, potentially resulting in adverse health outcomes [8].

Previous studies have found that the readability of existing EMs on various medical diagnoses exceeds the recommended reading level [5,9-11]. Because there is no specific method for assessing medical language, previous studies have used various readability-scoring methodologies. Herein, we investigate the readability of patient EMs and HPEMs from LLS, NCCN, and ACS using different readability formulas and describe the differences in readability scores.

## Materials and methods

We chose to use EMs from LLS, NCCN, and ACS based on our senior authors’ recommendations, as these websites are known to have accurate information for patients and physicians. EMs from LLS, NCCN, and ACS on various blood cancer diagnoses - acute myeloid leukemia, hairy cell leukemia, acute lymphoblastic leukemia, chronic lymphocytic leukemia, Waldenström macroglobulinemia, Hodgkin lymphoma, and non-Hodgkin lymphoma - were assessed for their readability using the following measures: the Flesch Reading Ease Formula (FREF), Flesch-Kincaid Grade Level (FKGL), Gunning Fog Index (GFI), Simple Measure of Gobbledygook Index (SMOG), and Coleman-Liau Index (CLI) [12-16]. FREF scores that correspond to sixth to seventh-grade reading levels are 70-90 [13]. For FREF, the closer the score is to 100, the easier it is to read [13]. FKGL, GFI, SMOG, and CLI scores approximately correspond to respective grade levels [12-16]. For example, a score of 12 means that the material is at a reading level of a twelfth-grader. We chose these readability formulas to obtain readability scores in various methods and to compare them because each readability formula uses different components, such as letters, words, sentences, and syllables. Also, these were chosen for ease of reporting in numerical scores, as some readability modalities are reported as graphs, which would need to be compared qualitatively.

We took all the documents from LLS, NCCN, and ACS and grouped them into either leukemia EMs, lymphoma EMs, or HPEMs. A total of 50 leukemia EMs, 42 lymphoma EMs, and 9 HPEMs were obtained from the aforementioned organizations’ websites. The materials were downloaded and converted to plain text to evaluate their readability. Images, tables and table captions, figures and figure captions, glossaries, indexes, and references were excluded (if present) to focus on the ability of readers to understand the main content. This way, the readability scores would be more accurate since some formulas include the number of sentences, and other less relevant texts, such as those previously mentioned, are not organized in a regular sentence structure. To avoid the readability scores from being miscalculated, texts besides the main content were excluded. Readability scores were obtained using the following formulas:

FREF = $206.835 - (1.015*(words/sentences)) - (84.6*(syllables/words))$

FKGL = $(0.39*(words/sentences)) + (11.8*(syllables/words)) - 15.59$

SMOG = $1.0430 * (\sqrt{total polysyllables*\frac{30}{total sentences}}) + 3.1291$

GF = $(0.4*(words/sentences)) + (100*(complex words/words))$

CLI = $0.0588L - 0.296S - 15.8$

where L = average number of letters per 100 words and S = average number of sentences per 100 words [12-16].

## Results

**Figure 1:**
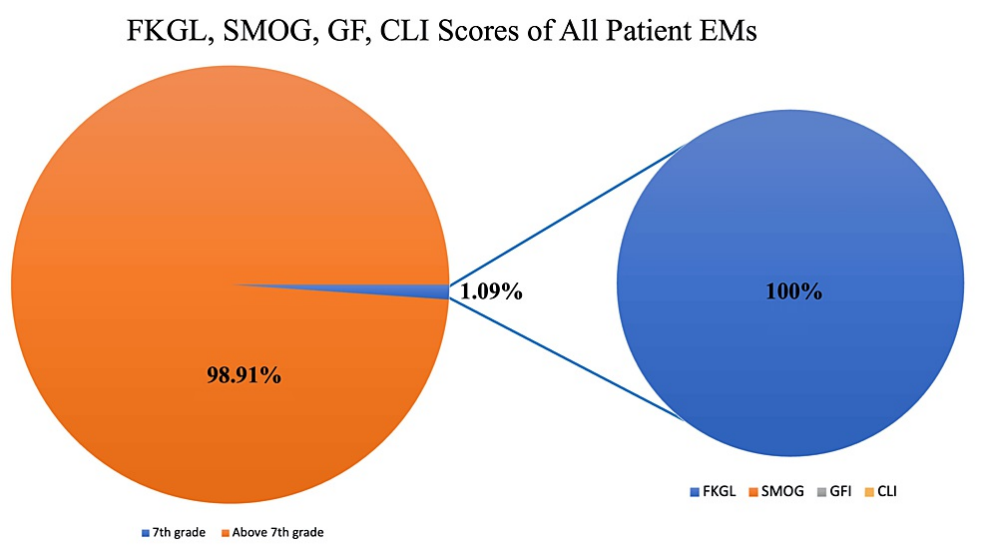
FKGL, SMOG, GFI, and CLI scores for all patient EMs Of all FKGL, SMOG, GFI, and CLI scores for patient EMs, only 1.09% scored at or below the seventh-grade reading level. Of the 1.09%, 100% were calculated by the FKGL formula. FKGL: Flesch-Kincaid Grade Level; SMOG: Simple Measure of Gobbledygook Index; GFI: Gunning Fog Index; CLI: Coleman-Liau Index; EMs: education materials

Of all FKGL, SMOG, GFI, and CLI scores for all EMs, only 0.99% scored at or below the seventh-grade reading level. Of all of the patient EMs, only 3.96% scored at or below the seventh-grade reading level when only considering the lowest score for each EM in any scoring modality (Figure 1). Leukemia patient EMs averaged higher than the sixth to seventh-grade reading level (FKGL = 9.60, GFI = 12.65, SMOG = 12.69, and CLI =11.91). The leukemia patient EMs had an average FREF score of 54.90. Lymphoma patient EMs averaged higher than the sixth or seventh-grade reading level (FKGL = 9.60, GFI = 12.73, SMOG = 12.74, and CLI = 11.85). The lymphoma patient EMs had an average FREF score of 54.90. HPEMs averaged around the college to graduate school reading level (FKGL = 14.63, GFI = 16.10, SMOG = 18.33, and CLI = 15.75).

Table 1 reports differences in the readability scores across different types of EMs (leukemia, lymphoma, or HPEM). HPEMs had consistently significantly higher readability than patient EMs for both leukemia and lymphoma, which supports our hypothesis.

**Table 1:** Average readability scores and standard deviations and t-test p values SD: standard deviation, * indicates a statistically significant difference (p<0.05) FKGL: Flesch-Kincaid Grade Level; SMOG: Simple Measure of Gobbledygook Index; GFI: Gunning Fog Index; CLI: Coleman-Liau Index; HPEM: healthcare professional education materials

Leukemia vs. Lymphoma			Leukemia vs. HPEM			Lymphoma vs. HPEM		
Leukemia FKGL Average (SD)	Lymphoma FKGL Average (SD)	p	Leukemia FKGL Average (SD)	HPEM FKGL Average (SD)	p	Lymphoma FKGL Average (SD)	HPEM FKGL Average (SD)	p
9.60 (1.70)	9.60 (1.08)	0.9871	9.60 (1.70)	14.63 (5.76)	0.0002*	9.60 (1.08)	14.63 (5.76)	0.0002*
Leukemia SMOG Average (SD)	Lymphoma SMOG Average (SD)	p	Leukemia SMOG Average (SD)	HPEM SMOG Average (SD)	p	Lymphoma SMOG Average (SD)	HPEM SMOG Average (SD)	p
12.65 (1.46)	12.73 (2.10)	0.7753	12.65 (1.46)	16.10 (3.76)	0.0006*	12.73 (2.10)	16.10 (3.76)	0.0006*
Leukemia GFI Average (SD)	Lymphoma GFI Average (SD)	p	Leukemia GFI Average (SD)	HPEM GFI Average (SD)	p	Lymphoma GFI Average (SD)	HPEM GFI Average (SD)	p
12.69 (1.25)	12.74 (1.26)	0.8555	12.69 (1.25)	18.33 (7.61)	0.0003*	12.74 (1.26)	18.33 (7.61)	0.0002*
Leukemia CLI Average (SD)	Lymphoma CLI Average (SD)	p	Leukemia CLI Average (SD)	HPEM CLI Average (SD)	p	Lymphoma CLI Average (SD)	HPEM CLI Average (SD)	p
11.91 (1.80)	11.85 (1.95)	0.8203	11.91 (1.80)	15.75 (4.39)	0.0005*	11.85 (1.95)	15.75 (4.39)	0.0003*

The results of differences between readability scoring modalities are reported in Table 2. Overall, FKGL scores are consistently significantly lower than SMOG, GFI, and CLI scores. SMOG and GFI scores were consistently similar and higher than CLI scores for leukemia and lymphoma EMs. In contrast, only GFI scores were consistently higher than both FKGL and CLI scores for HPEMs. SMOG and GFI scores were most consistent with each other. Our results show that the readability measures produce significantly different scores and suggest the readability of EMs may be inaccurate. However, as previously mentioned, the majority of patient EMs were above the seventh-grade reading level regardless of the type of formula used.

**Table 2:** T-test p values comparing readability scoring methods * indicates a statistically significant difference (p<0.05) FKGL: Flesch-Kincaid Grade Level; SMOG: Simple Measure of Gobbledygook Index; GFI: Gunning Fog Index; CLI: Coleman-Liau Index; HPEM: healthcare professional education materials; EMs: education materials

Type of EM	FKGL vs. SMOG p	FKGL vs. GFI p	FKGL vs. CLI p	SMOG vs. GFI p	SMOG vs. CLI p	GFI vs. CLI p
Leukemia Patient EMs	3.33E-21*	1.73E-22*	6.65E-14*	0.8464	0.0049*	0.0021*
Lymphoma Patient EMs	1.48E-18*	3.82E-22*	1.23E-12*	0.9752	0.0057*	0.0019*
HPEMs	0.1730	0.0078*	0.3077	0.0641	0.7196	0.0399*

## Discussion

Patient EMs from LLS, NCCN, and ACS averaged above the reading level recommended by the AMA and NIH. The results of our study are consistent with previous studies on education materials on various diagnoses across different specialties [5,9-11]. Although there were significant differences between the readability scoring modalities, the majority of patient EMs were above the seventh-grade reading level regardless of the scoring modality used. Healthcare professionals should be aware of the inconsistencies between readability scoring modalities when creating new and improving existing patient EMs.

SMOG and GFI scores were most similar among scoring modalities. Because there is no scoring modality specifically for medical language, it is important to highlight the differences and similarities between the available readability scores, as the choice of scoring modality may influence future patient and physician EMs. FKGF, GFI, and CLI scores were statistically significantly different (p<0.05) across leukemia EMs, lymphoma EMs, and HPEMs. Our findings were similar to those of the study performed by Grabeel et al. in 2018 in which SMOG scores were consistently significantly higher than FKGL scores for patient EMs [17]. However, our study found that the same was not true for HPEMs. Future goals to assess readability could include formulating a readability scoring method specifically for medical language.

In contrast to leukemia and lymphoma EMs, there were no significant differences in FKGL vs. SMOG, FKGL vs. CLI, SMOG vs. GFI, and SMOG vs. CLI for HPEMs. Specifically, only GFI scores were consistently higher than both FKGL and CLI scores. Possible explanations for this discrepancy include the possibility that readability scoring modalities (1) are more consistent for materials aimed at a higher reading level, (2) lose preciseness as reading level increases, or (3) are best suited up to grade 12, as post-high school education is highly variable. Healthcare professionals should be aware of these inconsistencies in readability scoring modalities when assessing EMs.

HPEMs were consistently appropriate at a graduate reading level, highlighting that medical communications are difficult to read due to their length and medical jargon. This, combined with the evidence of higher-than-recommended patient EM readability scores shows that translating technical medical language into layman’s terms may be a challenge. Difficult terminology could be combated with short informational videos, especially for chemotherapy-related terminology [18]. Given that more patients are looking to the Internet for healthcare information, existing patient EMs should be revised so that patients will be well-informed of their diagnosis and treatment options.

Leukemia is the most common pediatric cancer diagnosis [19]. It is important to aid parents in understanding their child’s disease and treatment options, especially in an era of rapid development of cancer care. In 2017, a study by Sinsky et al. reported that physicians spend 52.9% of their time on direct patient face time in the examination room with the patient in ambulatory settings [20]. Therefore, improving the readability of EMs targeted to the pediatric cancer patient population could be beneficial in educating the patient and the patient’s parents so that more time can be dedicated to discussing treatment options and expanding on the basic information already learned through EMs. A study on inclusive healthcare education by Koller in 2017 found that, when asked about knowledge of their medical conditions, pediatric patients wanted to learn more about their medical conditions and take part in the medical decision-making process [21]. Thus, it is important to create learning opportunities like easily understandable EMs for young audiences to fill in the gaps in their knowledge.

In the United States, cancer incidence is increasing in adolescents and young adults (AYAs), defined by the National Cancer Institute as anyone diagnosed with cancer between the ages of 15 and 39 [22-23]. For example, Hodgkin’s lymphoma is most common in this age cohort [24]. In 2017, a study by Domínguez & Sapiña reported that 80% of the AYAs in their cohort sought information about their diagnosis on the Internet and did not share that information with their parents or healthcare providers [25]. Another study reported that AYAs expressed a demand for specific medical, psychosocial, and healthcare-related education at the time of diagnosis and survivorship [26]. This shows that AYAs desire opportunities to improve their medical awareness to prioritize care and psychological maturation. Thus, EMs may serve as a source of coping and support for AYAs. Improving EMs will allow young patients to take control of their disease and empower them to be active participants in their treatment and care team.

“Chemobrain” or “chemo fog,” terms referring to the cognitive dysfunction caused by chemotherapy, can occur in up to 70% of cancer patients [27,28]. This potentially long-lasting condition manifests as memory loss, concentration issues, slowed speech, difficulty learning, inattention, coordination problems, and impaired executive functioning [27]. Doxorubicin, a chemotherapy drug commonly used for acute lymphoblastic leukemia, acute myeloblastic leukemia, and Hodgkin lymphoma, has been reported to cause this syndrome [28]. Chemo brain can negatively affect patients’ ability to learn new information about their diagnosis, which further emphasizes the importance of easily readable patient EMs. Such easily readable patient EMs should be available for all cancer patients, especially those experiencing chemobrain, outside of the clinical setting so that they can review these educational resources and learn at their leisure.

Key strategies for improvement involve using plain language, incorporating visual aids, considering cultural diversity, employing interactive formats, ensuring usability and accessibility, gathering feedback from patients, integrating information with healthcare providers, and regularly updating content. Recommendations to improve readability could include decreasing the number of words per sentence, utilizing words with less than three syllables when possible, and explaining medical jargon in simpler layman's terms.

A lack of easily readable patient materials could lead to a poor understanding of the disease, which ultimately increases the chances of adverse health outcomes that could be prevented with better educational resources [29]. If the reading level is higher than patients can understand, patients may seek information from other sources that may have incorrect information or misinformation that could influence the patient’s understanding of their disease and the recommended treatment. These materials should not replace physician counseling. It is imperative that physicians fill in the gaps in patients’ understanding of their diagnosis and treatment options throughout their cancer treatment.

To our knowledge, this is the first study to analyze the readability of leukemia and lymphoma patient EMs and HPEMs across five different readability modalities, compare the readability scores between patient EMs and HPEMs, and compare each EM across the five different measures. Our study has limitations in terms of scope. Our study was limited to the EMs available and published by ACS, LLS, and NCCN. Additionally, there was a limited number of HPEMs compared to that of patient EMs available across ACS, LLS, and NCCN. Furthermore, our study was limited to English EMs, and EMs of other languages available online may yield different results.

## Conclusions

Although nationally recognized by physicians as a valuable resource for cancer patients and survivors, our study found that current patient EMs available on LLS, ACS, and NCCN include complex and technical language higher than the recommended seventh-grade reading level. Although the readability measures produced varying readability scores, the scores were consistently above the seventh-grade reading level. Therefore, regardless of modality, these patient EMs may be difficult for an average American reader to fully understand. Difficult-to-read EMs may lead to poor health outcomes. Furthermore, standardization of a readability measure specifically for medical literature may be beneficial for ensuring a more uniform and accurate analysis of future and existing EMs.
